# A systematic review on lecturing in contemporary university teaching

**DOI:** 10.3389/fpsyg.2022.971617

**Published:** 2022-11-03

**Authors:** Héctor Tronchoni, Conrad Izquierdo, M. Teresa Anguera

**Affiliations:** ^1^Faculty of Teacher Training, University of Valencia, Valencia, Spain; ^2^Faculty of Education, Florida Universitària, Valencia, Spain; ^3^Faculty of Psychology, Autonomous University of Barcelona, Barcelona, Spain; ^4^Faculty of Psychology, Institute of Neurosciences, University of Barcelona, Barcelona, Spain

**Keywords:** systematic review, lecture, PRISMA, higher education, university teaching

## Abstract

**Introduction:**

Articles published in scientific journals, concerning the present and future of the lecture format in university education in the twenty-first century are framed within organizational settings that drive teaching methodologies in line with educational policies. The following two research questions have arisen from articles in which debate the continuity of this teaching modality and propose improvements of a different nature: (1) Is there an interest in renovating the lecture format among the international research community whose remit is university teaching methods? and (2) What improvements to the lecture format do the reviewed articles suggest, within the framework of the communicative matrix of interactive learning?

**Method:**

We have carried out a systematic review guided by the PRISMA approach, emphasizing the interest in methodological conceptual commitment, paying attention to documents published in journals with an impact factor. The search strategy was applied homogeneously in three databases: ERIC, PsycInfo, and Web of Science, following the systematic process of inclusion/exclusion.

**Results:**

Forty-five articles were selected with a range of 0–78 quotations, from different fields of knowledge and five continents; 12 articles are from journals with a JCR impact factor. The journal articles cover communicative (21), cognitive (13) and active-practical perspectives (11); the predominant governing aim of the analyzed improvements is connected with the attendees’ academic performance results (24); the reviewed studies belong mainly to the quantitative paradigm (42). The considerations derived from the results (45) cover formative, technical and/or critical aspects.

**Discussion and conclusions:**

Whilst positively valuing all these efforts promoted by the European Higher Education Area, we have also verified the lack of contributions in line with our concerns that embrace the need to develop an in-depth conceptualization, supported by a methodology that is sensitive to the complexity of the oral communication format between an expert actor and non-specialized actors who wish to connect and collaborate with the expert in the production of knowledge.

## Introduction

Within the context of the new vision of higher education (UNESCO Declaration, 1998; The Bologna Declaration, 1999) we propose to contribute to the renovation of teaching methodology by systematically reviewing the case of the university lecture format (Tronchoni et al., 2018, 2021; Tronchoni, 2019). We agree with the view that the expository-lecture format based on the programming of subject lessons should be reassessed both from a communicative standpoint and from the angle of the shared production of academic knowledge during university lessons.

The lecture is effectively one of the most used teaching methods in universities (Fortanet-Gómez and Ruíz-Madrid, 2014), and at first glance there does not appear to be an issue between the use of this teaching format and the institutional commitment to the development of democratic values and the promotion of social welfare. In fact, the study of the lecture as an improved expository format in higher education has its own place within the area of Instructional Communication within the field of interpersonal communication (Mazer and Hess, 2017).

In the last two decades there has been a proliferation of publications that deal with the lecture-type expository format (Pérez-Llantada and Ferguson, 2006; Deroey and Taverniers, 2011; O’Callaghan et al., 2017), noting the multiple functions and wide diversity of knowledge areas to which it is applied (Steinert and Snell, 1999; Dolnicar et al., 2009; Stacy, 2009; Tanahoung et al., 2009; Özcan, 2013), whilst highlighting the positive opinion that students have of this teaching format (Bates et al., 2017; Buchanan and Palmer, 2017).

Approval (or disapproval) of this teaching method ranges from emphasizing or questioning its effectiveness in small and large groups (Steinert and Snell, 1999; Kramer, 2017), to appraising the development of students’ listening and note-taking skills (Meyer and Hunt, 2017).

With the incorporation of active pedagogies in university teaching, different studies have shown a concern for the role played by the lecture in the students’ learning process (Barr and Tagg, 1995; Dannels, 2016; Darling, 2017; Tronchoni et al., 2021). This is giving rise to a change of direction in terms of understanding how active listening can benefit from other self-directed cognitive and emotional processes, whilst not forgetting the interpersonal communication skills that may mobilize the participants (Darling-Hammond et al., 2017; Mallin, 2017; Hayden and Chory, 2018; Stockard et al., 2018; Thwin and Lwin, 2018). Whilst it is assumed that the lecture is a face-to-face format, the incorporation of the Internet into formal teaching has led to the lecture format being increasingly present in different online educational modalities or synchronous hybrid contexts (Raes et al., 2020), with studies appearing concerning the use of interactive webinars (Gegenfurtner and Ebner, 2019) and pre-recorded lecture classes (O’Callaghan et al., 2017). Furthermore, over the last 2 years its synchronous virtual and online use has been propelled by the COVID pandemic (Younis and Elbanna, 2022).

This tendency has led to the publication of studies committed to the transformation of the lecture into what could be called the *new expert lecture*, a subject currently under debate within international higher education forums (French and Kennedy, 2017; Buzzanell, 2017; Darling, 2017; Meyer and Hunt, 2017; Sciullo, 2017; Stearns, 2017; Waldeck and Weimer, 2017; Samarasekera et al., 2018).

The synthetic review narrative that precedes the current situation of the lecture in higher education has led us to pose the central exploratory questions of this synthetic systematic review, reducing the PICO strategy to three elements: population (P), intervention (I), and result (O). Firstly, we seek answers to the following questions: (Research Question 1) Is there an interest in renovating the lecture format among the international research community whose remit is university teaching methods? And secondly (Research Question 2), what improvements to the lecture format do the reviewed articles suggest, within the framework of the communicative matrix of interactive learning (Ruesch and Bateson, 1951)?

## Method

This work follows the updated protocol of *The Preferred Reporting Items for Systematic reviews and Meta-Analyses* (PRISMA) for the transparent, complete and precise presentation of systematic review reports (Page et al., 2021).

### Search strategy

The search was carried out across three databases: PsycInfo, Web of Science, and ERIC, and the search strategy for identifying material was homogeneously applied: title containing the term *lecture*, AND *teaching methods* OR *lecture method* among the key words. This search tool was completed with the filters: articles in journals, university level education, peer review, complete text available, English language and time range from 2012 to 2021.

### Eligibility criteria

The eligibility criteria applied were: articles of an empirical nature applied to university level education, with a conventional summary and methodological structure (introduction, method, results, and discussion). The aim was to give priority to studies that deal with specific experiences of changes in the lecture, with a defined organization. Reviews of any kind (narrative, bibliographical or meta-analysis) were discarded on the assumption that the established period of analysis was insufficient to allow for the production of additional elaborative material.

### Selection process

**FIGURE 1:**
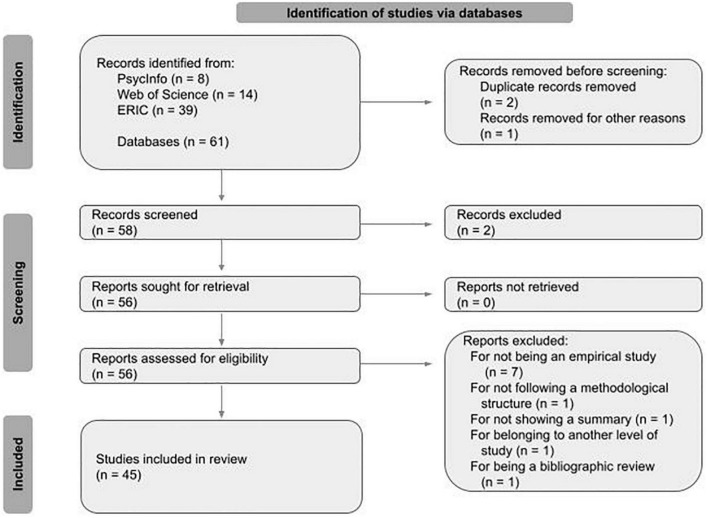
Flow diagram (PRISMA 2020) with selection process.

The selection process involved firstly a review of the titles and articles, and secondly a detailed review of the complete texts of the remaining articles taking into consideration the eligibility criteria (see Figure 1).

### Sample obtained

Table 1 includes all the selected articles (*n* = 45) in chronological order, with the year of publication, the name of the journal and the title of each article appearing from left to right.

**TABLE 1:** Selected articles in chronological order.

Year	Journal	Title
2012	Journal of Pragmatics	The interdependence of repetition and relevance in university lectures
2012	The Behavior Analyst Today	The relative effects of traditional lectures and guided notes lectures on university student test scores
2012	The Behavior Analyst Today	The Relation between GPA and Exam Performance during Interteaching and Lecture
2012	Interactive Learning Environments	Explaining students’ appraisal of lectures and student-activating teaching: Perceived context and student characteristics
2012	Biochemistry and Molecular Biology Education	Learning transferable skills in large lecture halls: Implementing a POGIL approach in biochemistry
2013	International Journal for the Scholarship of Teaching and Learning	Finding the Right Fit: Assessing the Impact of Traditional v. Large Lecture/Small Lab Course Formats on a General Education Course
2013	Advances in Language and Literary Studies	Developing a Specialized Vocabulary Word List in a Composition Culinary Course through Lecture Notes
2013	International Journal of Higher Education	Features of Application of Classroom Response System at the Lectures in Russia and Israel
2013	Novitas-ROYAL (Research on Youth and Language)	A Study on Perception of Lecturer-Student Interaction in English Medium Science Lectures
2013	Turkish Online Journal of Distance Education	A Comparison of Internet-Based Learning and Traditional Classroom Lecture to Learn CPR for Continuing Medical Education
2013	British Journal of Educational Technology	An augmented lecture feedback system to support learner and teacher communication
2014	Journal of the Scholarship of Teaching and Learning	Teacher Immediacy and Student Learning: An Examination of Lecture/Laboratory and Self-Contained Course Sections
2014	Teaching of Psychology	If you record it, some won’t come: Using lecture capture in introductory psychology
2015	Online Learning	Using Instructor-Generated Video Lectures in Online Mathematics Courses Improves Student Learning
2015	Informatics in Education	Using Short Video Lectures to Enhance Mathematics Learning–Experiences on Differential and Integral Calculus Course for Engineering Students
2015	Journal of Evolution of Medical and Dental Sciences-JEMDS	Comparison of the traditional chalk and board lecture system versus power point presentation as a teaching technique for teaching gross anatomy to the first professional medical students
2015	Journal of Interactive Media in Education	Digital Voting Systems and Communication in Classroom Lectures–An Empirical Study Based around Physics Teaching at Bachelor Level at Two Danish Universities
2015	Canadian Journal for the Scholarship of Teaching and Learning	Sustainability: Teaching an Interdisciplinary Threshold Concept through Traditional Lecture and Active Learning
2015	Journal of Evolution of Medical and Dental Sciences-JEMDS	Comparison of Problem Based Learning with Traditional Lectures among First Year Medical Students in Philosophy
2015	The Mathematics Educator	Research on Group Learning and Cognitive Science: A Study of Motivation, Knowledge, and Self-Regulation in a Large Lecture College Algebra Class
2016	International Journal of Higher Education	Integration of Histology Lectures and Practical Teaching in China
2016	Turkish Journal of Emergency Medicine	The comparison of the efficiency of traditional lectures to video-supported lectures within the training of the Emergency Medicine residents
2016	Journal of College Teaching & Learning	Preparing Students for Class: A Clinical Trial Testing the Efficacy between Multimedia Pre-Lectures and Textbooks in an Economics Course
2016	Journal of Curriculum and Teaching	The Use of Pre-Recorded Lectures on Student Performance in Physiology
2017	International Journal of Evaluation and Research in Education	Students’ Critical Thinking Improvement through “PDEODE” and “STAD” Combination in the Nutrition and Health Lecture
2017	Bali Medical Journal	Effectiveness of teaching: Jigsaw technique vs. lecture for medical students’ Physics course
2017	Advances in Engineering Education	Large Lecture Transformation: Improving Student Engagement and Performance through In-Class Practice in an Electrical Circuits Course
2017	GIST-Education and Learning Research Journal	Questions in English as a Medium of Instruction versus Non-English as a Medium of Instruction Lectures
2017	Journal of Education and Practice	The Effect of Instructional Methods (Lecture-Discussion versus Group Discussion) and Teaching Talent on Teacher Trainees Student Learning Outcomes
2018	Journal of Learning in Higher Education	Building a Case for Active Learning: The Use of Lecture vs. Other Classroom Activities at LMBC
2018	Computers & Education	Impact of slide-based lectures on undergraduate students’ learning: Mixed effects of accessibility to slides, differences in note-taking, and memory term
2018	Journal of Chiropractic Education	Comparison of student performance and perceptions of a traditional lecture course versus an inverted classroom format for clinical microbiology
2018	International Journal for the Scholarship of Teaching and Learning	Role-Play in Literature Lectures: The Students’ Assessment of Their Learning
2018	International Journal of Higher Education	Use of a Scaffolded Case Study Assignment to Enhance Students’ Scientific Literacy Skills in Undergraduate Nutritional Science Education: Comparison between Traditional Lecture and Distance Education Course Formats
2019	Journal of Learning Analytics	Diversity of Online Behaviours Associated with Physical Attendance in Lectures
2019	Anatomical Sciences Education	Interactive Lecture in the Dissection Hall: Transforming passive lecture into a dynamic learning experience
2019	English Language Teaching	Micro-Lecture Teaching for Improving the Learning Effect of Non-English Majors at North China Electric Power University
2019	European Journal of Contemporary Education	Three Scientific Facts about Ukrainian and Polish Law-Students: Verification of Statistical Hypotheses about their Preferences of Learning at Lectures
2019	International Review of Research in Open and Distributed Learning	Diversity in Video Lectures: Aid or Hindrance?
2020	Research in Learning Technology	The Effect of Adding Same-Language Subtitles to Recorded Lectures for Non-Native, English Speakers in E-Learning Environments
2020	International Journal of Higher Education	A UTAUT Evaluation of WhatsApp as a Tool for Lecture Delivery during the COVID-19 Lockdown at a Zimbabwean University
2020	Sage Open Nursing	Comparison of the Conceptual Map and Traditional Lecture Methods on Students’ Learning Based on the VARK Learning Style Model: A Randomized Controlled Trial
2020	Advances in Medical Education and Practice	Comparison Between Problem-Based Learning and Lecture-Based Learning: Effect on Nursing Students’ Immediate Knowledge Retention
2020	Bulletin of the University of Karaganda-Chemistry	Presenting lecture materials in English using CLIL technologies
2020	Journal of E-Learning and Knowledge Society	Does the sequence of flipped and lecture-based classes affect the academic achievement and satisfaction of medical students?

### Analytical framework

**FIGURE 2:**
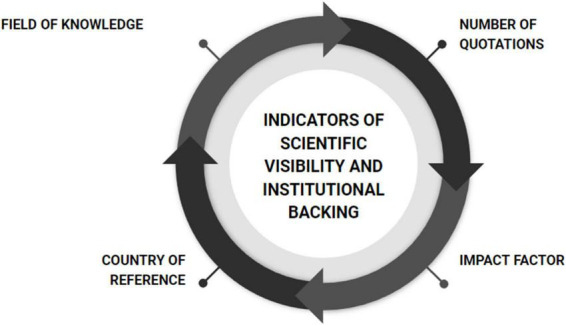
Criteria for the analysis of institutional backing and the visibility achieved with the publication of an empirical study aimed at improving the functioning of the university lecture.

The constructed analytical framework consists of two dataframes:

(I) Firstly, the scientific visibility and institutional backing of the selected research was coded (see Figure 2). The geographical origin indicator was taken into consideration since it provides information about the existing educational policies and quality demands in higher education in the universities of the countries of reference. Along with the country of reference, the knowledge area or discipline of the academic subject matter is indicated in those which generated some type of renewal proposal of the lecture format based on empirical evidence. The codes of the knowledge areas/disciplines are: sciences (CEX); biological sciences (BIO), medicine and health sciences (MED), social sciences (SOC), economic and business administration sciences (EAD), humanities (HUM) and diverse or indeterminate (DIV).

A distinction can be made between countries and continents, –and, ultimately, between universities and institutions that recognize the relevance of, or have financed, research in this field. This generates an ordered record of institutional recognition of the origin of said research. This data is completed by two relevance indicators of the knowledge produced and disseminated: we are referring to the number of quotations taken from the article, and the scientific evaluation received by the journal responsible for the publication from some of the most respected platforms concerning the assessment and analysis of performance and scientific research quality. The number of quotations is an indicator of the professional repercussion that the article has had, both in the area of university education and in the pedagogic and didactic research of teaching methods—this was obtained via Google Scholar and identified until the end of 2021; the positioning of the journal includes the impact factor (JCR-WoS) and the quartile (Q) to which it belongs according to the year the selected article was published.

(II) The second dataframe refers to the *multidimensional classification of the structural components* that produce significant differences in the way of conceiving and structuring the research object. As all the studies that make up the dataframe refer to how to relate and drive teaching and learning in the renovated use of the university lecture, we would like to point out that the practical and technical proposals do not always entail a theoretical justification identifiable as belonging to a recognized and named learning paradigm. Rather what is produced is a free use of concepts and techniques that can respond to different theoretical focuses (Entwistle, 2018). Taking this into account, the distinctive features considered were: conceptual perspective, guiding aim, type of study and applied result.

a) We identified three perspectives of a technical nature applied to the improvement of the lecture format: the communicative perspective (COM), the cognitive perspective (COG), and the participative-practical perspective (ACT).
b) The consolidated guiding aim that routed the selected research was conceived in terms of aptitude-treatment interaction (Cronbach and Snow, 1977) and the criteria derived to characterize the dominant concern were: student potential or aptitude (DIS), the strategies and conditions of the teaching to be carried out (INS), performance (REN) and the combination (COB) of criteria (INS-REN, INS-DIS, DIS-REN).
c) The methodological option that structures the research object on epistemological, ontological, and procedural levels can be specified with the widely argued and accepted proposal of quantitative (QUAN) and qualitative (QUAL) paradigms, and mixed-method (MM).
d) Finally, the applied results or conclusive recommendations can be understood as being aimed at assessing the education fostered by the lecture (FOR), technology for learning and knowledge (TEC), and the need to compare the use of the lecture with other teaching methods (CRI). These criteria can be presented combined in the same article (MIX).

Table 2 contains the symbols assigned to the categories used in the content analysis of the sample obtained via the PRISMA procedure.

**TABLE 2:** Dimensions of the analytical framework with components, symbols, categories, and examples for the analysis of the obtained data.

Dimensions of the analytical framework							
I. Scientific visibility and institutional backing			II. Multidimensional classification of the structural components of the analyzed research				
Component	Symbol	Category	Component	Symbol	Category	Example: Author, year/Fragment of article	
No of quotations (Google Academic)			Perspective	COM	Communication	Giménez-Moreno, 2012	Examine (.) the interdependence between relevance and repetition in current lecturing by firstly reviewing the main communicative strategies.
Impact factor JCR (sanctioning index of the relative relevance of the scientific journal depending on the quotations received)				COG	Cognition	Monk and Newton, 2018	Is the change in students SL skills related to their learning approach (i.e., deep versus surface learning approaches)?
Country (university) of reference relevance sanction				ACT	Active	Bailey et al., 2012	Keywords: active learning, cooperative/collaborative education
Field of knowledge/Studies	MED	Medicine and Health Sciences	Aim	REN	Performance	Kinnari-Korpela, 2014	Students’ learning is assessed
	SOC	Social Sciences: Education, Psychology		DIS	Dispositional	Starichenko et al., 2013	Reveal students’ attitude to CRS abilities
	CEX	Exact Sciences		INS	Instructional	Werner et al., 2018	Study about teachers
	BIO	Biological Sciences		COB	Combination	Chimmalgi, 2019	Students’ satisfaction and performance are assessed
	HUM	Humanities	Methodology	QUAN	Quantitative	Vázquez and Chiang, 2016	Controlled clinical experiment
	EAD	Economic and Business Administration Sciences		QUAL	Qualitative	Ní Riain et al., 2018	Qualitative research on the use of role-play
	DIV	Diverse or unidentified		MM	Mixed-Method	Navaz, 2013	Mixed-method perspective
Continent	AME	America	Result	FOR	Formative	Nordin et al., 2013	Title: Developing a Specialized Vocabulary Word List in a Composition Culinary Course through Lecture Notes
	EUR	Europe		TEC	Technological	Mathiasen, 2015	Title: Digital Voting Systems and Communication in Classroom Lectures
	ASI	Asia		CRI	Critical	Burnham and Mascenik, 2018	Title: Comparison of student performance and perceptions of a traditional lecture course versus an inverted classroom format for clinical microbiology
	AFR	Africa		MIX	Mixed	Drouin, 2014	Title: If You Record It, Some Won’t Come: Using Lecture Capture in Introductory Psychology
	ACE	Oceania					

## Results

Table 3 shows the analysis of the scientific production relevance indicators (Quotations and IF JCR) and the supported relevance in origin (country, university, disciplinary knowledge) of the reviewed empirical articles:

**TABLE 3:** Analytical framework of the sample of reviewed empirical articles.

No. of article	Frame of the scientific visibility and institutional backing				Multidimensional classification of structural components			
	No. of quotations	Impact factor	Country	Field of knowledge	Perspective	Guiding aim	Type of study	Results
1	78	0.70/Q4	USA	CEX	ACT	DIS	QUAN	FOR
2	77	–	USA	CEX	COG	REN	QUAN	TEC
3	73	1.394/Q1	Spain	SOC	COM	DIS	QUAN	TEC
4	53	–	USA	SOC	COG	INS REN	QUAN	FOR
5	52	–	Finland	CEX	COM	REN	MM	TEC
6	48	0.667/Q3	USA	SOC	COM	DIS REN	QUAN	TEC CRI
7	34	–	USA	SOC	COM	REN	QUAN	FOR
8	26	–	USA	SOC	COM	REN	QUAN	FOR
9	23	1.302/Q1	Belgium	SOC	ACT	DIS	QUAN	FOR
10	20	–	Sri Lanka	DIV	COM	INS DIS	MM	FOR
11	20	–	Iran	CEX	COM	REN	QUAN	FOR TEC
12	19	–	Turkey	MED	COG	REN	QUAN	FOR TEC
13	19	–	India	MED	ACT	REN	QUAN	FOR TEC
14	18	5.627/Q1	South Korea	SOC	COG	REN	QUAN	FOR TEC
15	16	–	Indonesia	BIO	ACT	REN	QUAN	FOR
16	15	–	Denmark	CEX	COM	REN	QUAN	TEC
17	15	–	USA	SOC	ACT	DIS	QUAN	FOR
18	12	–	USA	EAD	COG	REN	QUAN	FOR TEC
19	11	–	USA	MED	COM	DIS REN	QUAN	CRI
20	11	–	Russia/Israel	DIV	COM	DIS	QUAN	TEC
21	10	–	China	MED	COM	REN	QUAN	FOR
22	10	–	Australia	DIV	COM	DIS	QUAN	FOR TEC
23	9	–	Iran	CEX	COM	REN	QUAN	CRI
24	9	Q2	South Africa	HUM	COG	REN	QUAN	FOR TEC
25	9	–	Zimbabwe	DIV	COM	DIS	QUAN	TEC
26	8	3.759/Q1	India	MED	COG	DIS REN	QUAN	FOR
27	8	–	Malaysia	HUM	COM	INS	QUAN	FOR
28	7	2.297/Q1	South Korea	HUM	COG	REN	QUAN	FOR TEC
29	7	–	USA	CEX	ACT	DIS REN	QUAN	CRI
30	6	–	USA	SOC	COG	REN	QUAN	FOR
31	5	–	Poland/ Ukraine	SOC	COM	DIS REN	QUAN	FOR TEC
32	4	Q3	Iran	MED	COG	REN	QUAN	CRI
33	4	–	USA	CEX	COG	REN	QUAN	FOR
34	4	–	USA	EAD	ACT	INS	QUAN	FOR
35	3	0.7/Q2	Spain	EAD	COM	INS	QUAN	FOR
36	3	–	China	HUM	COM	REN	QUAN	FOR TEC
37	3	–	Spain	EAD	COM	INS	QUAN	FOR
38	2	–	USA	MED	ACT	REN	QUAN	FOR TEC
39	2	–	Indonesia	SOC	COM	REN	QUAN	CRI
40	2	–	Canada	MED	COG	DIS REN	QUAN	FOR
41	1	Q2	Ethiopia	MED	COG	DIS REN	QUAN	FOR
42	1	–	Kazakhstan	CEX	COM	REN	QUAN	FOR
43	1	–	India	MED	ACT	REN	QUAN	CRI
44	1	–	Ireland	HUM	ACT	REN	QUAL	FOR
45	0	Q3	Iran	MED	ACT	DIS REN	QUAN	CRI

### Scientific visibility and institutional backing

#### Number of quotations: presence/absence of links criterion

**FIGURE 3:**
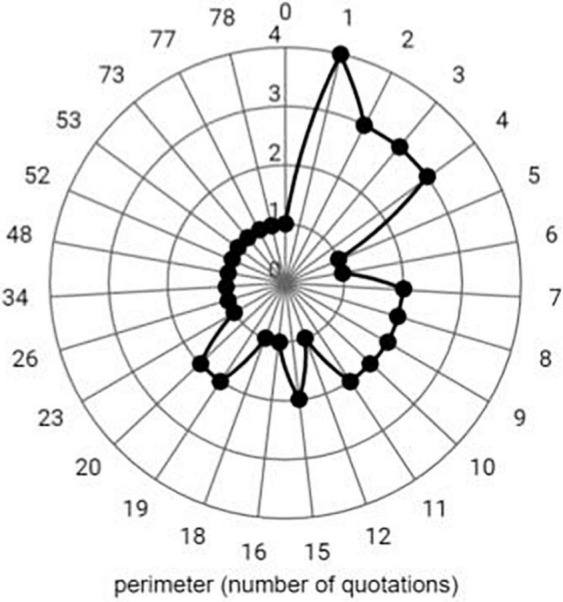
Relationship between number of quotations and number of articles.

The number of quotations ranges from 0, a study by Shabani et al. (2020), to 78 (see Figure 3), an article by Bailey et al. (2012) that deals with the transformation of the expository lecture in a large group, within a teaching format focused on the student. As the number of quotations rises, the number of articles diminishes, and therefore the most recently published articles show a lower number of quotations than the articles published in the first years of the time span used in this study (2012–2021). The number of accumulated quotations from all the articles as a whole was 759. It is worth assessing the presence of links among researchers concerned about the same issues rather than the quantity received, given that the materialization of synergies is more sensitive to the conditions in which the quotations are produced rather than the quantity of quotations received.

#### Impact factor: scientific reliability criterion

**FIGURE 4:**
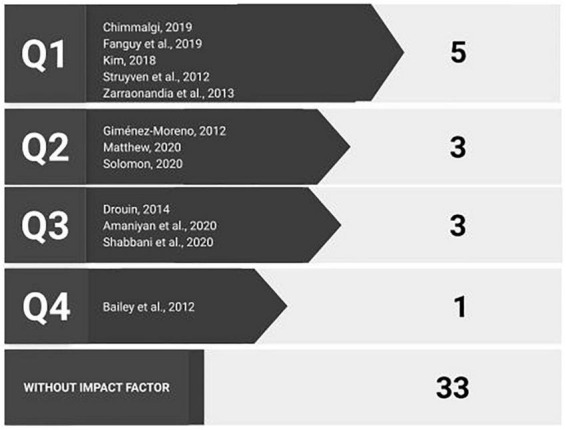
Placement of articles in journals with impact factor (JCR-WoS) and quartile (Q).

Of the 45 articles selected, 12 (27%) belong to journals with an impact factor: 5 of Q1, 3 of Q2, 3 of Q3, and 1 of Q4 (see Figure 4). The greatest impact factor is 5.627 and corresponds to an article published in the Q1 journal, *Computers & Education*, about the effects of access to projected slides during lectures using *Powerpoint* (Kim, 2018). The recognition that research groups and communities give to the need for an external assessment of their material before publishing, and the aspiration of being assessed by highly qualified journals, are two points that reflect the concern for obtaining applied results and a good path to achieving tangible applied results based on rigorous studies.

#### Geographical-academic distribution: institutional backing criterion

**FIGURE 5:**
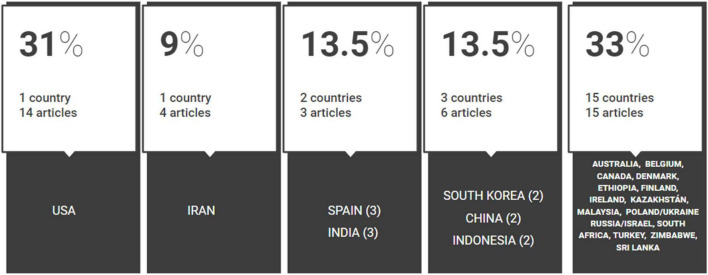
Geographical provenance ordered by number of articles.

The geographical distribution (see Figure 5) of the selected articles is presented, from highest to lowest incidence, as follows: USA (14), Iran (4), India (3), Spain (3), South Korea (2), China (2), Indonesia (2), and the rest of the 15 countries (1). The geographical distribution covers the continents: Asia (18), North America (15), Europe (8), Africa (3), and Oceania (1), and highlights the absence of articles from Central and South America. The diversity of countries and continents reflects the diversity of educational policies and proposals for the improvement of university education in the twenty-first century. However, the seminal ideas of a teaching founded on the attention to differences and on the proposal of active teaching methods with a vision tinged by constructivism and support for a spirit of collaboration, appear to emerge in the discourse of the international communities and groups dedicated to educational research within higher education. Since the UNESCO Declaration (1998) and the The Bologna Declaration (1999) on innovative educational methods, the lines of improvement converge on an intercontinental level in pedagogic and didactic terms. Another matter entirely is the availability of means (economic, equipment, teacher training, etc.) and the cultural codes involved in the regulation of the complex factors present in situations of interactive learning induced by expository and highly specialized formats of teaching, as is the case of postgraduate and doctoral lectures.

#### Field of knowledge: plasticity of innovative teaching methods criterion

In the group of selected articles, the studies deal with different areas of knowledge (see Table 4): MED (11), SOC (11), CEX (9), HUM (5), EAD (4), DIV (4), and BIO (1). Whilst all the curricular disciplines are involved in creating a renovation in the field of cognitive strategy communication, teamwork, or in the way of assessing results, the concern about improving the lecture format remains a didactic setting that can be adapted to interactive and collaborative learning conditions.

**TABLE 4:** Fields of knowledge of the analyzed studies.

Field of knowledge	Number of articles
MED	11
SOC	11
CEX	9
HUM	5
EAD	4
DIV	4
BIO	1
**Total**	**45**

### Multidimensional classification of structural components

The articles cover three perspectives: COM (21), COG (13), and ACT (11). The plasticity of the didactic methods shown above facilitates a complex approach to the research object. The limits are imposed by the research object as defined by the researcher, although the frequency of the studies assigned to one category or another is not the most important thing. What we wish to highlight is that the three orientations are present in the analyzed sample.

The guiding-aim of the selected studies correspond to: INS (4), DIS (7) and REN (24) and in 10 articles two of these aims are combined: INS + REN (1), INS + DIS (1), and DIS + REN (8). One sign of lecture maintenance is precisely that responses are designed to the problems of performance, and to those arising from teacher conduct as being responsible for the teaching action involved in all the facilitating modes of interactive learning.

The methodologies used in the generation and analysis of data focus on the QUAN perspective (42), while the other two options are only present in three articles (QUAL, 1 and MM, 2). It is important to underline that the journals with a higher impact factor located in the quartiles Q1 and Q2 do not propose any methodological restrictions, whilst some of them even advocate openly for the inclusion of research that responds to the quantitative, qualitative and mixed-method approaches. These journals are: *Anatomical Sciences Education, British Journal of Educational Technology, Interactive Learning Environments, Computers & Education, International Review of Research in Open and Distributed Learning, Research in Learning Technology, Journal of Pragmatics, Advances in Medical Education and Practice*.

The applied results and/or recommendations from the studies influence the following aspects: FOR (20), CRI (7), TEC (6), and in 12 articles two of these are combined: FOR + TEC (11) and TEC + CRI (1). What stands out is the teaching value of the lecture and the incorporation of telematic computerized resources. It also provides a reflection about the role of technical aspects in pedagogic and didactic improvements.

The intersection of the conceptual perspective dimension with the methodological affiliation provides us with a new picture of the data when the nine resulting regroupings are considered (see Table 5).

**TABLE 5:** Conceptual-methodological commitment (perspectives and methodologies).

Perspective conceptual/ Methodology	COM	COG	ACT	Total
QUAN	3, 6, 7, 8, 11, 16, 19, 20, 21, 22, 23, 25, 27, 31, 35, 36, 37, 39, 42	2, 4, 12, 14, 18, 24, 26, 28, 30, 32, 33, 40, 41	1, 9, 13, 15, 17, 29, 34, 38, 43, 45	**42**
QUAL			44	**1**
MM	5, 10			**2**
Total	**21**	**13**	**11**	**45**

An analytical view of the methodological commitment in each regrouping is presented as a whole in Table 6, characterized by:

**TABLE 6:** Analytical view of the methodological commitment in each regrouping.

Methodological commitment	No of articles	No of quotations	Impact factor					Field of knowledge							Continent					Aim				Result			
			Q1	Q2	Q3	Q4	S F I	S O C	C E X	M E D	H U M	E A D	B I O	D I V	A M E	A S I	E U R	A F R	O C E	R E N	D I S	I N S	C O B	F O R	T E C	C R I	M I X
QUAN-COM	19 (42.2%)	301 (39.7%)	1	1	1	0	16	6	4	2	2	2	0	3	4	8	5	1	1	9	4	3	3	7	4	3	5
QUAN-COG	13 (29%)	220 (29%)	3	2	1	0	7	3	2	5	2	1	0	0	6	5	0	2	0	9	0	0	4	6	1	1	5
QUAN-ACT	10 (22%)	165 (21.7%)	2	0	1	1	6	2	2	4	0	1	0	0	5	4	1	0	0	4	3	1	2	5	0	3	2
QUAL-ACT	1 (2.3%)	1 (0.1%)	0	0	0	0	1	0	0	0	1	0	0	0	0	0	1	0	0	1	0	0	0	1	0	0	0
MM-COM	2 (4.5%)	72 (9.5%)	0	0	0	0	2	0	1	0	0	0	1	1	0	1	1	0	0	1	0	0	1	1	1	0	0
**Total**	**45 (100%)**	**759 (100%)**	**6**	**3**	**3**	**1**	**32**	**11**	**9**	**11**	**5**	**4**	**1**	**4**	**15**	**18**	**8**	**3**	**1**	**24**	**7**	**4**	**10**	**20**	**6**	**7**	**12**

#### Methodological commitment QUAN-COM, QUAN-COG, and QUAN-ACT

There are 19 QUAN-COM articles (42.2%) and they include 301 quotations (39.7%). Three articles have an impact factor (Q1, Q2 and Q3), and are from diverse fields of knowledge: SOC (6), CEX (4), DIV (3), EAD (2), MED (2); are from different continents: AME (4), ASI (8), EUR (5), AFR (1), and OCE (1). The aims are of type REN (9), DIS (4), INS (3) and DIS-REN (3), and the results of type FOR (7), TEC (4), CRI (3), TEC-CRI (1) and FOR-TEC (4).

There are 13 QUAN-COG articles (29%) and they include 220 quotations (29%). Six articles have an impact factor (Q1, Q2, and Q3), and are from diverse fields: MED (5), SOC (3), CEX (2), HUM (2), and EAD (1); and are from different continents: AME (6), ASI (5), and AFR (2). The aims are of type REN (9), DIS-REN (3), and INS-REN (1), and the results are of type FOR (6), FOR-TEC (5), TEC (1), and CRI (1).

There are 10 QUAN-ACT articles (22%) and they include 165 quotations (21.7%). Four have an impact factor (Q1, Q3, and Q4), are from diverse fields of knowledge: MED (4), CEX (2), SOC (2), and EAD (1), and are from three continents: AME (5), ASI (4) and EUR (1). The aims are of type REN (4), DIS (4), DIS-REN (2), and INS (1), and the results are of type FOR (5), CRI (3), and FOR-TEC (2).

To summarize, the presence of the quantitative conceptual-methodological commitment in all the other structural aspects considered, and its prevalence in the most accredited scientific media, leads us to the conclusion that the web of quotations could provide interesting results for the subjects they cover.

#### Methodological commitment QUAL-ACT

One QUAL-ACT article was identified, with one quotation and no impact factor. It is from Ireland, and the field of knowledge is HUM. The aim is of type REN and the result is FOR.

In light of this datum and taking into account the comment about the methodological aperture of the journals with a JCR impact factor, it is worth underlining the numerous possibilities offered by qualitative methodology (Wertz et al., 2011).

#### Methodological commitment MM-COM

There are 2 MM-COM articles; they include 72 quotations (9.6%) and have no impact factor. The fields of knowledge are CEX and BIO, and they are from Europe and Asia. The aims are REN in one article and INS-DIS in the other, and the results are TEC and FOR.

Given that we believe the mixed-method conceptual-methodological commitment is suitable for the study of the innovative renovations of the lecture format (Tronchoni et al., 2021), and the scientific works with methods considered as inherent integrators of qualitative and quantitative data analysis (Anguera and Izquierdo, 2006; Bazeley, 2018; Izquierdo and Anguera, 2021), we can only hope that the MM commitment will be present in more studies. On the other hand, the products achieved with this conceptual-methodological commitment are appreciated, valued and recommended for publication in the best placed journals that cover the subjects of teaching and learning in higher education.

## Discussion and conclusions

A descriptive analysis of the results obtained via the coding of the criteria dimensions in order to capture the differences produced by the systematically selected sample, produces two conclusions that provide answers to the two research questions posed in the introduction: the first conclusion responds to the question about the current scientific situation of the renovated lecture format subjected to empirical study. The second conclusive response places value on the identified processes and results, whilst at the same time demonstrating the need to articulate a proposal that incorporates an open and dialogued vision of the teaching system, whose continued renovation should be founded on empirical research in as far as this is possible and necessary.

Understanding the evolution of the relationships between the various components of the teaching system is vital if universities are to offer effective and efficient teaching. Given that we have found no other systematic reviews of the proposed key terms, it is not possible either to verify whether the description carried out and the conclusions we propose are in line with other reviews, nor to indicate in which aspects our findings differ from those provided by other systematic reviews of the lecture format.

Before entering into the argumentation thread that sustains the inferential and proactive path of this section, it is worth pointing out that our empirical conceptual-methodological approach connected with educational assessment (Tronchoni et al., 2021) has a point of contact—differences aside—with the *Direct Instruction* movement (Engelmann and Colvin, 2006). When it comes to discussing the coarse matter of the frequency distribution of the different criteria and the subtle silence of the vacuums that the analyzed results produce, we include as a contrast the idea of systematizing the way the acquisition of new knowledge is accessed in terms of direct instruction proposals, i.e., the teaching system of the lecture in our case. We believe that the vacuums or lowest scores can provide a certain generalization of interest about the need to construct a common base open to a plurality of viewpoints, but with a clear message about the need to systematize the lecture format without renouncing flexibility, plasticity, web connectivity, or sustainable effectiveness.

Research Question 1. Is there an interest in renovating the lecture format among the international research community whose remit is university teaching methods?

Conclusion 1. The geographical channeling of institutional backing and the evaluation of the visibility and scientific reliability of the web of quotations is proof positive for tackling the internationalized challenge of the renovation of the lecture format. Unfortunately, the methodological commitment remains incomplete in not providing qualitative and mixed-method studies, and the databases consulted are not sensitive to the research carried out in South American countries.

This conclusion is based on the following evidence found in our research:

a) In the group of selected articles, the lecture is present in a wide diversity of journals. Some of them show a JCR impact factor, elaborated on the *Web of Science* platform (WoS). The visibility of this subject in the scientific-academic community interested in the renovation and innovation of teaching methods in general and the lecture in particular, is guaranteed in the period consulted. The five journals with the highest impact factor according to year of publication, in ascending order are: *Interactive Learning Environments* (2012), *British Journal of Educational Technology* (2013), *International Review of Research in Open and Distributed Learning* (2019), *Anatomical Sciences Education* (2019), and *Computers & Education* (2018). The conceptual-methodological commitments QUAN-COM (3), QUAN-COG (6), and QUAN-ACT (3) were present in the articles with an impact factor. The articles conceived as QUAL-ACT (1) and MM-COM (2), were published in journals indexed on other platforms.
b) The expository lecture, with a greater or lesser scientific visibility, is a subject that raises interest among researchers in different countries on all continents. The geographical prevalence of the selected articles belongs to the scientific production of researchers in Asia (mainly Iran, India, South Korea, China and Indonesia) and the USA. Our database search did not produce selected articles from South America. On the other hand, Asia and North America cover the methodological commitments QUAN-COM (12), QUAN-COG (11), and QUAN-ACT (9); Europe shows an interest in the methodological commitment QUAN-COM (5) and Africa in QUAN-COG (2) and QUAN-COM (1). Once again, the options that structure the research object with a methodological commitment QUAL or MM were silenced.
c) The appearance of quotations in the scientific production of articles is an indicator of the appreciation and value given to the subject, and of its subsequent incorporation into new articles that promote the applied proposals in real situations within the field of the acquisition of curricular, declarative and procedural knowledge, and of that pertaining to the area of values, attitudes, and emotions. Quotations generate networks of interest through the mobilization of said advances, creating tendencies within studies. This suggests that networks of influence are being formed. The expository lecture is defined by a directly visible web potential that has set in motion 759 connections in the total of the 45 articles in our sample. Only one recently published article did not register any quotations. The web is structured into lesser groupings depending on the methodological commitments found from the QUAN commitment and these are thus distributed from greater to lesser number of quotations: QUAN-COM (301), QUAN-COG (220), and QUAN-ACT (165).

Research Question 2. What improvements to the lecture format do the reviewed articles suggest, within the framework of the communicative matrix of interactive learning?

Conclusion 2. It is very difficult to know the characteristics of the lectures that have been subject to intervention and their relationship with other teaching and learning methods. However, the internationalized agenda of the subjects covered is sufficiently pertinent to give rise to partial improvements in the exploitation of technological opportunities (ICT) applied to the transmission of knowledge, the use of strategies and the inclusion of participative tasks and techniques. Unfortunately, the analyzed sample does not reflect the concept of a communicative matrix within the organizational and institutional context of the intervened lectures; whilst all the articles are in line with the ideal of promoting *interactive learning*, the consideration as to how teaching should be adjusted does not appear.

The reflection of the obtained data on the improvements achieved by the interventions carried out in lectures covers the following subjects:

a) *Information processing and performance.* A total of 17.7% of the higher education sample analyzed describes teaching based on improvements in lecture design, beginning with the problems raised by providing information to be effectively remembered (DIS-REN, 8). The subject of attention and memory functions in interactive learning is linked to good performance and an increase in learning potential in lectures (French and Kennedy, 2017). Another aspect to bear in mind when considering the effects of the informative approach proposed to the students is whether to promote learning based on investigative competencies or on repetitive production activities (Lundvall and Johnson, 2016). Since both forms of learning are complementary, there is room for the design of mixed trajectories. The shadow of unmonitored (by the teacher) repetitive learning grows longer when we consider that 53% of our sample places emphasis on performance (REN, 24). We do not know the diversified cultures of the universities that use the lecture method, nor do we know the relationship between this format and other methods applied in class sessions, but the emphatic concern about performance might be indicative of a more conventional (the exclusive performance of declarative content) rather than innovative feature of the researched teaching practice.
b) *Prevalent thematic resonance.* Two articles with greater resonance show the polarities of the professional and research interests of the studies carried out.
  On the one hand, the article by Bailey et al. (2012) with IF (JCR) 0.70 (Q4) has 78 quotations (29% of the group of articles with an impact factor). This article was published in the journal *The International Union of Biochemistry and Molecular Biology*, by three professors from the Biochemistry Department of the University of Nebraska and the Chemistry Department of Seattle University (USA). The article deals with how to transform an expository lecture into a format centered on the students’ learning. The professional nature of the proposal connects with the pedagogical revolution of renovating the lecture by incorporating opportunities for participation (ACT), and developing skills (DIS) that are necessary for the learning of content that can be transmitted with an expository format (FOR).
  On the other hand, the article by Hegeman (2015), of Missouri Western State University (USA), has 77 quotations (16% of the group of articles with no impact factor) and was published in the journal *Online Learning*. The article analyzes the results of learning facilitated by the use of multimedia material and note-taking. The students are given cards designed by the teacher in order to guide the study of pre-recorded expository algebra sessions. The article, conceived from a cognitive perspective (COG), promotes the implication of the teaching staff in the technical handling (TEC) of telematic opportunities and the construction of tutorial material, with the aim of facilitating a greater performance (REN) from the students.
c) *Communicative matrix of the educational system.* It is striking that just 13% of the sample gives a description of teaching that places the focus on the teaching format in relation to performance or student aptitude (INS-REN, 1; INS-DIS, 1), and on the appropriateness of the lecture for carrying out certain learning (INS 4). One silenced aspect derived from this fact is the concern about the influence of teacher-student learning relationships (Entwistle, 2018). The good design and good application of an educational system—which may be the improved lecture—should incorporate the complex loop of communicative interactions between the four identifiable levels of exchange in the educational system: (i) the political-economic and cultural-educational conditions both inside and outside the classroom, (ii) the academic and professional side of the disciplines, (iii) the physical and virtual meeting spaces, and (iv) the personalities of the students and teachers. This reading of the renovated lecture reinforces the ritual of roles (Goffman, 1967) that can promote dialogic interaction within the juncture of teaching with interactive learning. There are those who search for this juncture in valuing the role played in the design and execution of the lecture by the active, attentive student who is a synthesizer of retrievable and revisable information. Another line of juncture seeks to potentiate oral participation. In this context of considerations, 71% of the reviewed studies direct their concern about communication toward academic performance, with some mentions of student aptitude or the expository teaching of the content covered (COM-REN, 10; COM-COB, 4). If we take into account that 47% of the articles incorporate the COM perspective, then it would be important for the subject of academic performance to predominate in order to vouch for the virtues of the renovated lecture format. We suspect that the communicative focus does not respond to a holistic and dialogic approach, and that the concern about individual student aptitude is centered on the satisfaction that preferably cognitive experiences generate in being able to drive learning itself, with the aid of the teacher’s exposition and the development of necessary strategies and abilities.
d) *The geographical and institutional extent of attempts to improve the lecture format does not provide the necessary contextualization of those innovations.* The internationalization of the aim of improving the lecture format, understood as an expert teaching format, is undoubtedly benefiting from the possibilities offered by ICT, multimedia options and the combined use of face-to-face and synchronous virtual sessions (O’Callaghan et al., 2017; Gegenfurtner and Ebner, 2019; Raes et al., 2020). These new possibilities, sensitive to the educational and economic conditions of each university and country, present the need to develop specific designs in terms of how to implement and drive instructional participative interaction that mobilizes reasons to cooperate, together with the strategic use of collaborative cognitive-emotional abilities implied in the process of acquiring academic knowledge.

The reviewed articles propose changes (COM, COG, ACT) but do not give contextual keys to understanding how to go about it and to conceptualize what is proposed as an intellectual and experiential result above and beyond a mere recuperation of information.

To summarize, from standpoint regarding the conceptualization of the innovation of the lecture format and the conceptual-methodological commitment adopted in the empirical research of this subject (Tronchoni et al., 2021), we believe that the review carried out alerts us to the lack of studies that provide an integrated response to the conditions and roles of interactive learning, together with tackling the production of emotional-intellectual experiences that reinforce the dialogic and collaborative links of all the participants. Whilst all the methodological options might be appropriate for structuring empirical studies about the improvements sought by good interactive design and a good execution of the renovated lecture, we would like to underline that the mixed-method approach of systematic observation (Anguera et al., 2017) fits well with the idea of being able to finalize reliable formative assessments contingent upon the diversity of people, the disciplines involved, space-time conditions, own and imported educational cultures, and the most distal influences. The focus of the observation centers on participative interaction, a mechanism responsible for the organization of exchanges and for controlling the means of producing academic knowledge, and for the emotional-intellectual experience. Above and beyond the satisfaction produced by academic results, the emotional-intellectual experience that emerges from the social implication in the construction of knowledge can be considered a powerful resource for personal growth and collective wellbeing (Claxton, 1984).

Every systematic review has inherent limitations to its own profile—such as the proposal of selection criteria for primary documents—that inevitably have an influence on the results, both on those obtained, and on the vacuums detected. An example of this is the *culture of research* itself which may exist in relation to the expository lecture in Latin American countries, made invisible by opting for selection criteria that we feel to be suitable (such as the English language). Furthermore, another limit could be that the filter with the term *lecture* may hide diverse understandings of the lecture format within the specification of each study.

## Data availability statement

The original contributions presented in this study are included in the article/supplementary material, further inquiries can be directed to the corresponding author.

## Author contributions

All authors listed have made a substantial, direct, and intellectual contribution to the work, and approved it for publication.
